# Dynamic Risk Inference Method for Chemical Industrial Inspection Based on Spatio-Temporal Scene Graphs

**DOI:** 10.3390/s26134082

**Published:** 2026-06-27

**Authors:** Meng Zhou, Liheng Wang, Sai Li, Zhixia Ding

**Affiliations:** School of Electrical and Information Engineering, Wuhan Institute of Technology, Wuhan 430205, China

**Keywords:** state estimation, mobile inspection, heterogeneous scene graph, kinematic-aware anisotropic dynamic field, uncertainty-aware adaptive hysteresis filtering, process safety

## Abstract

**Highlights:**

**What are the main findings?**

- A kinematic-aware anisotropic dynamic field transforms rigid geometric boundaries into deformable risk gradients to suppress spatial observation ambiguities.
- An uncertainty-aware adaptive hysteresis filter dynamically adjusts state machine thresholds according to real-time sensor noise, which reduces the false alarm rate to 1.3 times/h.

**What are the implications of the main findings?**

- The mathematical decoupling of spatial observation blur and temporal viewpoint jitter effectively overcomes the “alarm chattering” bottleneck inherent in dynamic mobile inspections.
- The low-latency processing capability (24.8 ms/frame) satisfies the stringent requirements for highly reliable edge-computing deployment in industrial process safety monitoring.

**Abstract:**

To address the challenge of high false alarm rates caused by dynamic viewpoint noise in mobile chemical inspections, this study established a highly robust adaptive dynamic risk inference model. This research proposes an inference framework integrating spatio-temporal semantic constraints. Spatially, this study constructed a heterogeneous dynamic scene graph and introduced a kinematic-aware anisotropic dynamic field. This field transforms geometric hard boundaries into continuous risk gradients that deform dynamically with target intentions to suppress observation ambiguity. Temporally, the work designed an uncertainty-aware adaptive hysteresis filter, whose state machine thresholds adjust dynamically according to real-time sensor noise levels. Comparative tests on a real-world chemical dataset show that the model achieves a peak F1-Score of 93.1%, reduces the false alarm rate to 1.3 times/h, and requires a single-frame processing time of only 24.8 ms. The method theoretically achieves spatio-temporal dynamic noise reduction, significantly mitigates topological mutations and alarm chattering under complex visual noise conditions, meets edge computing deployment requirements, and provides a high-confidence sensing decision hub for industrial process safety monitoring.

## 1. Introduction

Chemical production environments feature characteristics such as high temperature, high pressure, flammability, and explosiveness. Utilizing autonomous mobile robots equipped with visual perception systems to conduct all-weather intelligent inspections has become an inevitable trend to enhance the intrinsic safety of process industries [1,2,3]. However, in large complex industrial environments, multi-source environmental interference and dynamic non-stationary noise continuously restrict the large-scale application of inspection systems. Existing visual inspection systems primarily rely on static object detection networks, which present a severe “semantic gap” and fail to infer the interaction intentions and spatial semantic associations between targets. In recent years, Scene Graph Generation (SGG) technology was introduced into industrial visual cognitive frameworks [4,5,6]. However, when applied to mobile inspections, it faces the challenge of spatiotemporal topologies being highly susceptible to fracture and mutation under dynamic observation viewpoints [7]. Furthermore, static detection lacking temporal smoothing logic easily triggers frequent transient false alarms when encountering local occlusions and visual distortions. Meanwhile, dynamic temporal models that attempt to introduce black-box architectures, such as cascaded deep networks, often exhibit limited generalization in long-tail conditions due to a lack of explicit spatial physical constraints, thereby subjecting operators to severe “alarm fatigue” [8,9,10]. Recently, transformer-based temporal reasoning and Temporal Graph Neural Networks (TGNNs) have shown strong capabilities in capturing long-range dependencies in video anomaly detection [11,12]. However, deploying hybrid industrial perception models that rely on heavy self-attention mechanisms onto power-constrained edge robots remains computationally prohibitive. Furthermore, these data-driven black-box models often lack explicit geometric priors, making them vulnerable to domain shifts when the physical layout of the chemical plant changes.

To address the aforementioned issues, this study moved beyond the traditional logic of relying solely on visual network stacking to propose a dynamic risk inference model integrating spatio-temporal semantic constraints. First, a heterogeneous dynamic scene graph and a semantic projection mechanism were constructed. This study combined camera pose transformations to project global prior high-risk areas as virtual nodes to achieve unified graph-theoretic modeling. Secondly, this work constructed a kinematic-aware anisotropic field constraint model. This study discarded the traditional static Gaussian potential field and introduced the personnel movement velocity vector captured by visual sensors into the field strength calculation. This enables the risk response gradient to undergo dynamic spatial deformation alongside personnel movement intentions to effectively resolve the discrimination ambiguity caused when targets wander parallel to the edge of hazardous zones. Finally, this research designed an uncertainty-aware adaptive hysteresis ST-FSM. This study introduced a visual sensor confidence and continuous frame jitter evaluation mechanism to enable the hysteresis threshold of the state machine to undergo adaptive width adjustment according to the environmental non-stationary noise level. This endows the early-warning decision with an exceptionally strong high-frequency anti-chattering capability at the mathematical level.

To clearly distinguish the proposed framework from existing methodologies in industrial monitoring and scene graph generation, Table 1 summarizes the core differences in spatial modeling, temporal logic, and noise suppression mechanisms.

## 2. Architecture of the Chemical Mobile Inspection Visual System

**Figure 1:**
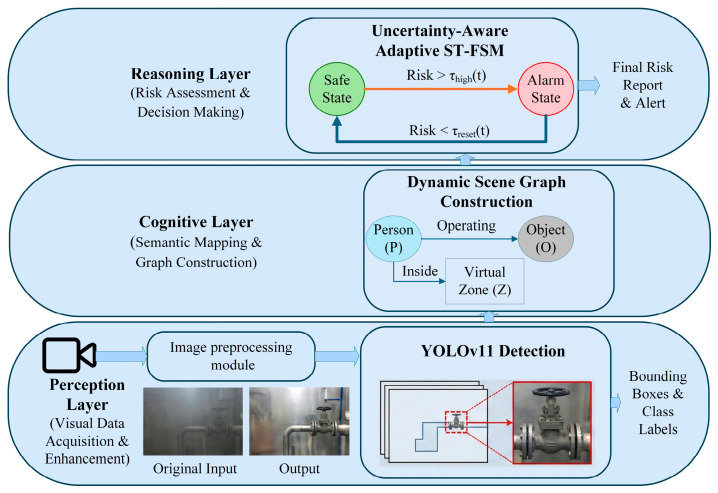
Overall architecture of the visual system for chemical inspection.

To address the unstructured environments, dynamic viewpoint changes, and multi-source multimodal interference faced in mobile chemical park inspections, this study moved beyond the traditional approach of relying solely on hardware sensors to construct a hierarchical and progressive “perception–cognition–decision” intelligent visual early-warning framework, as shown in Figure 1. The architecture aims to serve as an intelligent edge node for the chemical park HSE (Health, Safety, and Environment) management system to bridge the gap from low-level visual information acquisition to high-level process safety risk inference. The overall system architecture comprises three logical layers from bottom to top. The perception layer acts as the “visual soft measurement hub” of the system, responsible for the quality restoration of raw video streams and the refined detection of key equipment and personnel under complex and harsh operating conditions (e.g., low illumination, smoke interference). The cognitive layer serves as the “cognitive hub”, responsible for mapping discrete detection boxes into dynamic scene graphs containing topological relationships to complete the transformation from pixel space to industrial semantic space. The reasoning layer acts as the “decision hub”, conducting long-term sequential logical analysis on scene semantics based on spatio-temporal finite-state machines to output the final risk assessment results. The structured risk alert information output by this architecture directly integrates into the DCS (Distributed Control System) or emergency command platform of the chemical park with low latency to achieve closed-loop management with cloud-edge collaboration.

## 3. Theoretical Models and Methods

Traditional detection algorithms often exhibit jumps due to environmental noise when applied to mobile devices. This study transformed inspection risk early-warning into a “system state estimation under noisy observation” problem to construct a robust three-layer architecture encompassing perception, mapping, and inference.

### 3.1. Construction of Heterogeneous Dynamic Scene Graphs and Semantic Explicitization

To uniformly describe the complex “entity interaction” behaviors and abstract “spatial constraint” conditions in chemical scenarios, this study proposed a construction method for the heterogeneous dynamic scene graph $G_{t}=(V_{t},E_{t})$. Distinct from traditional scene graphs containing only visually observable targets, this structure introduced virtual environment nodes to transform the hard constraints of geometric space into soft connections within the graph topology to achieve a unified modeling of the physical world and the semantic space.

To solve the problem that traditional scene graphs solely focus on visual foreground entities (such as personnel and equipment) while neglecting background environment constraints, this research proposed an “environmental semantic explicitization” strategy. The work defined the heterogeneous node set $V_{t}=V_{phy}\cup V_{env}$. Specifically, $V_{phy}=\{v|v\in \{P,O\}\}$ corresponds to the physical entities detected by the visual perception layer, including personnel nodes P possessing active behavioral capabilities and equipment nodes O (such as valves and instruments) acting as operational objects. $V_{env}$ represents abstract environmental entities to characterize spatial areas within the chemical park possessing specific safety attributes (such as the high-pressure zone $Z_{high}$ and the toxic zone $Z_{toxic}$).

Unlike physical nodes generated directly by visual detectors, environmental nodes serve as logical anchors mapped to the current visual plane via perspective projection transformation based on the prior semantic map and localization information of the robot. Virtual environment nodes essentially represent artificially delineated static high-risk attribute domains within the global three-dimensional physical space. Under monocular or binocular visual systems, robots cannot directly “see” these invisible boundaries. Therefore, this study proposed a “semantic explicitization” projection strategy.

**Figure 2:**
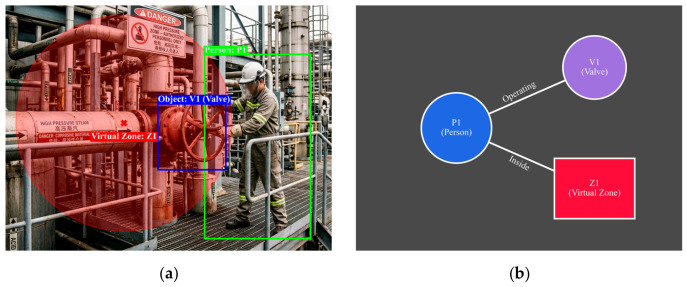
Construction example of the heterogeneous dynamic scene graph: (**a**) video frame with detections; (**b**) corresponding heterogeneous scene graph. (Note: The non-English characters on the warning signs are the direct Chinese translations of the accompanying English text, such as “High Pressure Steam” and “Authorized Personnel Only”).

During the actual inspection and mapping process, the robot obtained the real-time pose state of the current camera through the onboard SLAM (Simultaneous Localization and Mapping) system. Perspective transformation was applied to the three-dimensional spatial geometric center coordinates $P_{w}=[X_{w},Y_{w},Z_{w}{]}^{T}$ of the high-risk areas preset in the global semantic map to dynamically project them onto the two-dimensional visual image plane of the current robot to generate logical anchors $p_{c}=(u,v)$ within the visual mapping domain:

(1) $$z_{c}\left[\begin{array}{c} u\\ v\\ 1 \end{array}\right]=K\left[R|t\right]\left[\begin{array}{c} X_{w}\\ Y_{w}\\ Z_{w}\\ 1 \end{array}\right]$$

where K denotes the camera intrinsic matrix, [R|t] denotes the real-time extrinsic matrix (rotation and translation vectors) of the camera at the current moment, and $z_{c}$ denotes the depth scale factor. The logical anchor $p_{c}$ acts as the “virtual environment node” in the heterogeneous scene graph to enable the invisible hazardous boundaries in the physical space to obtain precise two-dimensional coordinate placeholders on the image plane. Through this perspective projection transformation formula, the system automatically calculates and updates the dynamic positions of environment nodes in the visual space with frame-level real-time performance during the mobile inspection process of the robot to completely replace traditional manual zoning and achieve automated semantic mapping from the three-dimensional physical world to the two-dimensional cognitive space, as shown in Figure 2.

It is worth noting that in real-world mobile deployments, the inherent cumulative drift and mechanical vibrations of the SLAM odometry inevitably introduce transient errors to the extrinsic matrix (ΔR,Δt). According to Equation (1), these dynamic 6DoF errors will directly translate into high-frequency translation and scale jitter of the projected logical anchor $p_{c}$ on the 2D image plane. This explicit geometric projection vulnerability strictly necessitates the subsequent uncertainty-aware temporal filtering design to prevent severe “alarm chattering” near the boundaries.

### 3.2. Scale-Adaptive Interaction and Kinematic-Aware Anisotropic Soft Constraint Modeling

To address the semantic ambiguity between “proximity” and “operation” caused by depth loss under monocular vision, this study proposed a hierarchical semantic mapping strategy. The generation criterion function F:V×V→E for the semantic edge set $E_{t}$ were divided into two abstract levels: “physical entity interaction” and “environmental space subordination”. This work modeled these levels using deterministic geometric constraints and probabilistic field theory constraints, respectively.

At the physical interaction level, the study introduced a scale-adaptive geometric constraint based on human pose estimation to accurately determine whether personnel actually contacted or operated the equipment. Recent studies indicate that YOLO-Pose maintains excellent keypoint localization accuracy and real-time performance when facing complex occlusions in industrial operating environments by introducing an improved Transformer architecture into the backbone network [13,14]. This research extracted the personnel hand keypoint set $H_{p}$, the equipment operation area center pixel coordinates $c_{o}$, and the equipment bounding box width and height $W_{o},H_{o}$ output by the visual perception layer. The system determines a strong interaction relationship (Operating, $e_{hold}$) when the keypoints satisfy the following conditions:

(2) $$e_{hold}\left(v_{p},v_{o}\right)=I\left(\underset{h\in H_{p}}{min}||h-c_{o}|{|}_{2}<\tau_{contact}\right)$$

where I(⋅) denotes the indicator function, $\tau_{contact}=\gamma_{hold}\cdot max(W_{o},H_{o})$ denotes the scale-adaptive threshold, and $\gamma_{hold}$ denotes the tolerance coefficient. This normalized discrimination design based on the dimensions of the bounding box itself effectively overcomes the extreme reliance on absolute dynamic camera ranging values and the scale sensitivity issues of traditional rule-based discrimination methods.

The weak interaction relationship (Near, $e_{near}$) characterizes the spatial proximity between entities. Similarly, the study utilized a relative threshold adapting to the personnel bounding box dimensions to execute the determination:

(3) $$e_{near}\left(v_{p},v_{o}\right)=I\left(||c_{p}-c_{o}|{|}_{2}<\tau_{prox}\right)$$

where $c_{p}$ denotes the personnel node center coordinates, and $\tau_{prox}=\gamma_{near}\cdot max(W_{p},H_{p})$ denotes the dynamic proximity threshold. This relationship typically serves as a preceding state for strong interactions to capture non-standard approaching or loitering behaviors.

At the environmental subordination level, the study cross-disciplinarily introduced the Virtual Potential Field theoretical concept widely adopted in mobile robot dynamic path planning [15,16,17]. In the traditional static Gaussian potential field, the risk gradient exhibits an isotropic distribution and neglects the movement intentions of targets. To portray the actual risk of personnel approaching hazardous sources more accurately, this research proposed a kinematic-aware anisotropic dynamic field constraint model.

**Figure 3:**
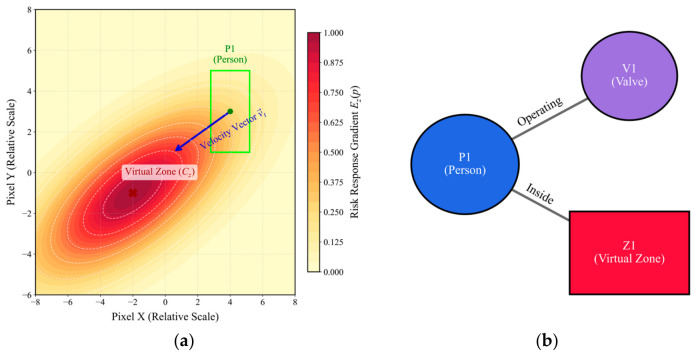
Mapping of heterogeneous scene graph and kinematic-aware anisotropic dynamic field. (**a**) kinematic-aware anisotropic potential field; (**b**) heterogeneous dynamic scene graph.

Let $p_{t}$ denote the personnel center coordinates captured by the visual sensor at time t. The study obtained the movement velocity vector $v_{t}=p_{t}-p_{t-1}$ on the pixel plane through inter-frame differencing. The work defined the risk field function of the environmental node z for the personnel $p_{t}$ as

(4) $$E_{z}\left(p_{t}\right)=\alpha_{z}\cdot \exp\left(-\frac{1}{2}(p_{t}-C_{z}{)}^{T}\Sigma_{t}^{-1}(p_{t}-C_{z})\right)$$

where $\alpha_{z}\in (0,1]$ denotes the inherent regional hazard coefficient; $C_{z}$ denotes the logical anchor coordinates corresponding to the hazardous zone. The core innovation of the kinematic-aware anisotropic dynamic field lies in the real-time construction of the covariance matrix $\Sigma_{t}$. Unlike static isotropic fields, $\Sigma_{t}$ dynamically stretches in the direction of the target’s velocity vector $v_{t}=[v_{x},v_{y}{]}^{T}$. Let the scalar speed be $s=||v_{t}||$, and the orientation angle of the movement be $\theta =arctan(v_{y}/v_{x})$. The covariance matrix is formulated through an eigenvalue decomposition framework:

(5) $$\Sigma_{t}=R(\theta )\left[\begin{array}{cc} \lambda_{1} & 0\\ 0 & \lambda_{2} \end{array}\right]R(\theta {)}^{T}$$

where R(θ) is the 2D rotation matrix aligning the primary axis with the velocity vector. The eigenvalues $\lambda_{1}$ and $\lambda_{2}$ control the variance along the moving direction and its perpendicular axis, respectively, defined as $\lambda_{1}=\sigma_{base}^{2}\cdot (1+\rho \cdot s)$ and $\lambda_{2}=\sigma_{base}^{2}$. Here, $\sigma_{base}$ represents the base safety penalty radius, and ρ is the velocity scaling coefficient. To guarantee mathematical stability and avoid singularity when the target is stationary or moving extremely slowly (s<ϵ, where ϵ→0), the field smoothly degrades to a standard isotropic Gaussian distribution, ensuring $\Sigma_{t}$ remains strictly positive-definite under all kinematic conditions. This mechanism transforms the originally rigid geometric ranging into a continuous gradient response containing kinematic intentions to greatly buffer the decision jumps caused by viewpoint perturbations. This research defined the global spatial risk input for any target $p_{i}$ in the field of view as Equation (6). The system utilizes this global risk value for subsequent temporal confidence integration and state machine inference, and the spatial mapping result of this field is illustrated in Figure 3.

(6) $$U_{s}(p_{i}{)}_{t}=\underset{z\in V_{env}}{max}E_{z}\left(p_{t}\right)$$

### 3.3. ST-FSM Uncertainty-Aware Adaptive Hysteresis Filtering and State Inference

In modern chemical process control, frequent invalid alarms severely increase the cognitive load on control room operators, which causes “alarm fatigue” and even triggers severe accidents (referencing the ISA-18.2/IEC 62682 [18] alarm management standards). To eliminate decision jumps caused by instantaneous target occlusion or viewpoint jitter, this study constructed a temporal confidence integration mechanism possessing first-order Markov properties. Cumulative risk state $B_{t}$ of the target was defined at time t, which depended not only on the spatial risk input $U_{s}(p_{i}{)}_{t}$ at the current moment but also, recursively, on the historical state $B_{t-1}$. The work defined its state update equation as

(7) $$B_{t}=\lambda \cdot B_{t-1}+(1-\lambda )\cdot U_{s}(p_{i}{)}_{t}$$

where λ∈[0,1] denotes the memory factor. Equation (6) essentially constitutes a discrete-time digital low-pass filter, which effectively filters out transient high-frequency feature noise. For instance, when personnel rapidly cross a hazardous zone, although $U_{s}$ reaches a high level momentarily, the cumulative value $B_{t}$ fails to rise quickly to the alarm threshold under the smoothing effect of λ due to the short duration, thereby filtering out the instantaneous false alarm. Conversely, when personnel continuously linger, the continuous input of spatial risk causes $B_{t}$ to accumulate gradually and approach the saturation value, exhibiting an S-shaped curve characteristic similar to capacitor charging, and thereby accurately capturing authentic violation behaviors.

Based on the cumulative risk value $B_{t}$ and the scene graph semantics, this study designed a two-state finite state machine (ST-FSM) to determine the final alarm behavior. This research defined the state set S={Safe,Alarm}. Considering that the reliability of single-frame observation is highly non-stationary in authentic industrial visual sensing, the anti-chattering capability of traditional fixed-threshold hysteresis comparators is severely limited when encountering extreme sensor noise (such as large-area smoke occlusion or strong mechanical jitter). Therefore, this research proposed uncertainty-aware adaptive hysteresis filtering.

**Figure 4:**
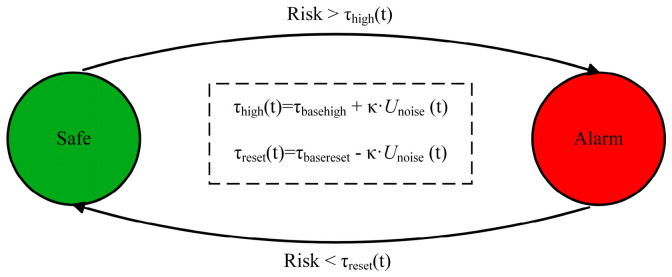
State transition logic diagram of the uncertainty-aware adaptive ST-FSM. (Note: The dashed box contains the dynamic calculation formulas for the warning threshold and reset threshold).

To quantify this, this study defined the uncertainty index $U_{noise}(t)\in [0,1]$, which is explicitly quantified by fusing spatial bounding-box jitter and semantic confidence variance. Let ${\mathrm{IoU}}_{t,t-1}$ denote the Intersection over Union of the target’s bounding boxes between consecutive frames, and $\sigma_{c}^{2}$ represent the variance in the classification confidence scores, calculated over a sliding temporal window of size W. The formulation is defined as

(8) $$U_{noise}(t)=min\left(1.0,\omega_{1}(1-{\mathrm{IoU}}_{t,t-1})+\omega_{2}\left(\frac{\sigma_{c}^{2}}{\sigma_{max}^{2}}\right)\right)$$

where $\omega_{1}$ and $\omega_{2}$ are normalized weighting factors ($\omega_{1}+\omega_{2}=1$), and $\sigma_{max}^{2}$ is the empirical maximum variance used for normalization. This formulation ensures that $U_{noise}(t)$ is strictly bounded. Subsequently, this work reconstructed the fixed hysteresis thresholds into dynamic functions varying over time with this calculated sensor uncertainty:

(9) $$\tau_{high}\left(t\right)=\tau_{base_{high}}+\kappa \cdot U_{noise}\left(t\right)$$

(10) $$\tau_{reset}\left(t\right)=\tau_{base_{reset}}-\kappa \cdot U_{noise}\left(t\right)$$

where $\tau_{base_{high}}$ and $\tau_{base_{reset}}$ denote the base thresholds, and κ denotes the adaptive gain coefficient. The parameter κ controls the sensitivity of the hysteresis expansion. Extensive sensitivity analysis indicates that setting κ ∈ [0.15, 0.25] provides an optimal balance: it prevents the hysteresis band from becoming excessively wide (which would cause missed alarms during authentic hazards) while sufficiently absorbing extreme visual uncertainty. The system updates the state transition function δ based on the real-time dynamic thresholds. The physical and mathematical significance of this mechanism lies in the following. When the environment remains stable ($U_{noise}$ approaches 0), the hysteresis band maintains a normal width to guarantee high sensitivity for early warnings. When the environment deteriorates and visual detection boxes jitter violently ($U_{noise}$ increases significantly), the system automatically widens the hysteresis band (increasing $\tau_{high}$ and decreasing $\tau_{reset}$) to adaptively strengthen the “anti-chattering” armor of the system at the mathematical level, as illustrated in Figure 4. This effectively mitigates the practical engineering problem of unreliable state estimation under mobile viewpoints.

*Local Stability Proof:* Assume the system currently operates in the Alarm state (i.e., $B_{t-1}\geq \tau_{high}$), and the visual observation suffers from interference by high-frequency positioning noise $v_{t}$ with a mean of 0 and a variance of $\sigma_{v}^{2}$. If the system adopts a single-threshold mechanism without hysteresis, when the authentic spatial risk decreases to near $\tau_{high}$, minimal ranging disturbances cause the system state to oscillate violently between Alarm and Safe (the chattering phenomenon). After introducing the hysteresis mechanism, the condition for the system to maintain the Alarm state relaxes to $B_{t}>\tau_{reset}$. To force the system to undergo an erroneous state reversal, the transient noise disturbance amplitude must overcome the hysteresis margin $\Delta \tau =\tau_{high}-\tau_{reset}$. By setting parameters reasonably to ensure $\Delta \tau >3\sigma_{v}$, the system obtains asymptotic anti-interference capability against local high-frequency jitter from the mathematical mechanism, which establishes strong theoretical bounds for the industrial-grade smoothness of the early-warning decision logic and provides a robust countermeasure for unreliable state estimation under mobile viewpoints.

## 4. Experimental Validation

### 4.1. Experimental Dataset and Experimental Environment

To verify the robustness of the model in authentic complex industrial environments, this study constructed the Chem–Industrial–Real (CIR) evaluation dataset oriented towards chemical scenarios. This research collected data from a large modern coal chemical park, and the scenes comprehensively covered typical high-risk areas such as the “high-pressure reactor zone”, the “intricate pipe gallery inspection channel”, and the “central control instrument room”. The dataset annotated seven core targets and the anomalies faced during mobile inspections: Person, Helmet, Valve, Fire, Smoke, Leak, and Rust.

To ensure high-quality ground truth, the annotation protocol followed a strict multi-annotator consensus mechanism: three domain experts independently labeled the sequences, and bounding boxes were only accepted if the Inter-Annotator Agreement (IAA) exceeded an IoU of 0.85. The dataset comprises a total of 125 continuous video sequences. The temporal distribution of anomalous events ranges from brief transient occurrences (e.g., a 2 s fast pass) to long-term violations (e.g., 45 s unauthorized operations), with an average anomaly duration of 14.3 s. Specifically, the 125 sequences are explicitly distributed across the three highly challenging sub-scenarios: 45 sequences in the high-pressure storage tank area, 50 in the intricate pipe gallery, and 30 in the central control room. Regarding class distribution, the dataset contains over 28,000 instance annotations. The majority consist of standard industrial entities such as Person (42%), Valve (25%), and Helmet (15%), while critical risk events like Fire, Smoke, Leak, and Rust account for the remaining 18%. This severe class imbalance faithfully reflects the typical long-tail distribution characteristics of authentic chemical industrial anomalies.

To ensure the evaluation data possessed sufficient time spans and statistical significance, the original data originated from inspection videos lasting 200 min. Through sparse sampling and keyframe extraction at fixed time intervals (1 frame/second), the dataset ultimately contained 12,000 annotated high-definition images (1920 × 1080), which the study divided into a training set (60%), a validation set (20%), and a test set (20%). To simulate highly challenging unstructured conditions in authentic inspections, the test set specifically included the following three high-difficulty subsets. Subset-A consisted of images captured during robot movement, containing significant motion blur and viewpoint jitter. Subset-B contained samples where personnel were severely occluded by densely intertwined process pipelines and protective guardrails. Subset-C contained extreme lighting conditions such as high brightness (direct sunlight) and low light (nighttime inspection). Specifically, the high-pressure storage tank area exhibited intense lighting changes, large metal tank occlusions, and yellow warning lines on the ground. The valve pipeline area featured dense pipelines, narrow spaces, and key valve handwheels. The data collection equipment utilized industrial cameras (Hikvision Digital Technology Co., Ltd., Hangzhou, China)(resolution 1920 × 1080).

This experiment aimed to verify the effectiveness and robustness of the proposed risk inference model through a two-stage testing approach: offline workstation equipped with an Intel Core i7-14650HX CPU (Intel Corporation, Santa Clara, CA, USA) and an NVIDIA RTX 4080 GPU (NVIDIA Corporation, Santa Clara, CA, USA) evaluation and online edge physical deployment. For the offline evaluation and model training, the platform consisted of a workstation equipped with an Intel Core i7-14650HX CPU and an NVIDIA RTX 4080 (24 GB) GPU, running the Ubuntu 20.04 (Canonical Ltd., London, UK) operating system with the PyTorch framework (version 2.1.0). To rigorously address the gap between offline algorithmic evaluation and real-world industrial deployment, the validation was further extended to an online edge-computing framework. The complete intelligent visual early-warning pipeline was compiled using TensorRT (version 8.x.x, please check your actual version) (NVIDIA Corporation, Santa Clara, CA, USA) (FP16 precision) and deployed on an NVIDIA Jetson AGX Orin (NVIDIA Corporation, Santa Clara, CA, USA) (64 GB) embedded development board. This board served as the edge AI node mounted on a wheeled intelligent inspection vehicle, operating under realistic industrial power and thermal constraints.

During the model training phase, considering that abnormal samples in chemical parks exhibit a “long-tail distribution” and limited quantities, this study strategically introduced a transfer learning mechanism to avoid the overfitting risk caused by small samples. Specifically, the YOLOv11 detector was pretrained on the COCO dataset to acquire generalized visual feature extraction capabilities and subsequently fine-tuned it on the CIR training set. Simultaneously, the study jointly utilized data augmentation strategies during the training process, including Mosaic stitching, random erasing, and dynamic blur simulation, which greatly enhanced the generalization capability of the model when dealing with occlusion and viewpoint jitter.

In authentic industrial cloud-edge collaborative deployment, the generation of virtual environment nodes relies on automated mapping. The edge-side robots carry multi-line LiDAR and inertial measurement unit (IMU) modules to run high-precision SLAM algorithms (such as LIO-SAM) to construct three-dimensional point cloud semantic maps. During dynamic inspections, the system utilizes the 6DoF pose calculated by the real-time odometry to map three-dimensional hazardous zones into two-dimensional logical anchors in real-time using Equation (1). In addition, in independent physical validations on edge-side computing boxes (such as NVIDIA Jetson AGX Orin), the system only needs to upload the extracted lightweight topological logs (in JSON format, approximately 2–5 KB/frame) to the cloud DCS system. Compared to transmitting 1080 P video streams directly, the communication bandwidth requirement plummets by 99.8%, which confirms the practical feasibility of this cloud-edge architecture from the engineering physical link.

However, given that the core experiments in this section proceeded based on the 12,000-frame CIR offline visual dataset, the system could not directly access online physical SLAM data streams. To ensure the engineering authenticity of the evaluation and restore the aforementioned automated projection process, this study adopted an “equivalent simulation” strategy during the testing phase. Based on the typical positioning drift characteristics of wheeled robots operating in actual factory areas, Gaussian random perturbations were artificially injected with a standard deviation of $\sigma_{n}\approx 15∼30$ pixels on top of manually calibrated ideal logical anchor coordinates. This equivalently simulates the dynamic projection jitter generated by authentic SLAM odometers in complex terrains. The comparative tests conducted under this stringent setting objectively reflect the anti-noise robustness of each model in authentic physical deployments.

### 4.2. Evaluation Metrics

To comprehensively evaluate model performance, this study introduced the F1-Score, defined as the harmonic mean of Precision and Recall:

(11) $$F1=\frac{2\times Precision\times Recall}{Precision+Recall}$$

Oriented towards the spatio-temporal risk inference task, this research specifically introduced two key metrics: False Alarm Rate (FAR) and Jitter Robustness Score (JRS). The study defined FAR as the frequency of the system erroneously triggering alarms under risk-free conditions per unit time (times/hour), which directly reflects the suppression capability of the model against dynamic noise. This research defined JRS as the probability of the system output state maintaining consistency (i.e., $P(State_{t}=State_{t-1})$) when the robot remains stationary but slight positioning drifts exist near boundaries. Additionally, to evaluate the computational efficiency of the algorithm, the work compiled statistics on the average processing time of a single frame image (inference time, in ms), which included the entire process of image enhancement, object detection, pose estimation, scene graph construction, and inference.

### 4.3. Comparative Experimental Results and Analysis

To verify the effectiveness of the proposed method while ensuring the fairness and rigor of comparative experiments, the study unified all comparative methods to employ YOLOv11s as the underlying feature extractor and established four sets of comparative baselines. Baseline 1 (pure spatial hard decision) executes instantaneous alarms based solely on bounding box overlap (IoU > 0), lacking scene graphs and temporal logic. Baseline 2 (hard threshold decision based on Euclidean distance) executes hard threshold decisions based on keypoints and absolute physical distances (possessing relationship constraints, lacking potential fields, lacking temporal logic). Baseline 3 (LSTM-Seq end-to-end) connects an LSTM network after the detector to perform end-to-end temporal classification (possessing temporal modeling, but lacking explicit scene graphs and spatial constraints, utilized to verify the advantages of explicit topologies over implicit black boxes). Baseline 4 (MotifNet static scene graph) [19] adopts a classic scene graph network to extract topological relationships and relies on simple threshold alarms (possessing scene graph spatial constraints, lacking state machine temporal filtering). Baseline 5 (ST-Transformer) introduces a modern spatio-temporal transformer architecture for video anomaly detection. It utilizes self-attention mechanisms to capture long-range temporal dependencies but essentially operates as a data-driven black box without explicit physical scene constraints. This baseline is utilized to benchmark the trade-off between the high accuracy of complex attention mechanisms and the computational efficiency required for edge devices. The proposed method (Ours) directly adopts the lightweight YOLOv11s-Pose network to output pose information without significantly increasing the computational load while integrating heterogeneous scene graph construction and spatio-temporal inference.

A comprehensive performance comparison of models under the various sub-conditions of the CIR test set is shown in Table 2. Experimental results show that detection relying solely on instantaneous visual features (Baseline 1, 2) collapses extremely easily under dynamic conditions, with overall False Alarm Rates (FAR) reaching as high as 18.2 and 8.5 times/hour, respectively. Although Baseline 3 realizes smooth filtering at the temporal level by introducing LSTM (FAR decreases to 2.3 times/hour), it struggles to utilize physical geometric priors to conduct effective inference when facing Subset-B (complex pipeline occlusion) due to the lack of explicit spatial topological constraints. This causes its F1-Score to be only 80.3%, and the intensive tensor operations of the end-to-end temporal network increase the single-frame inference time to 35.6 ms. Meanwhile, although Baseline 4 introduces structured scene graphs to enhance spatial constraints, its FAR remains elevated (4.1 times/hour) when facing the short-term losses of node features caused by lighting and viewpoint jitter due to the absence of state machine filtering in the temporal dimension. Furthermore, while the advanced Baseline 5 (ST-Transformer) achieves competitive F1-Scores (up to 89.0%) by leveraging heavy self-attention layers, its severe computational overhead drastically pushes the single-frame inference time to 110.5 ms, rendering it impractical for low-power edge robots. In contrast, the proposed method (Ours) demonstrates outstanding performance on all challenging subsets through the joint decoupling effect of anisotropic dynamic field soft constraints and state machine hysteresis integration. The method compresses the overall FAR to 1.3 times/hour and achieves a JRS of up to 92.3%. On the high-performance desktop workstation, the complete single-frame inference—encompassing YOLOv11s-Pose detection, scene graph construction, and ST-FSM state estimation—required only 24.8 ms. More crucially, to validate its practical feasibility, the system was tested on the NVIDIA Jetson AGX Orin edge node. Under actual hardware constraints, the overall end-to-end processing latency stabilized at 42.5 ms/frame (approximately 23.5 FPS). This hardware-in-the-loop performance securely surpasses the 15 FPS industrial threshold, fully substantiating the low-latency edge computing claims for autonomous dynamic inspections.

The *Inference Time* reported in the table reflects the baseline performance on the workstation (RTX 4080). Real-world edge physical deployment of the proposed framework on the Jetson AGX Orin achieved an average latency of 42.5 ms/frame, successfully maintaining real-time processing capabilities.

### 4.4. Ablation Experiment Results and Analysis

**Figure 5:**
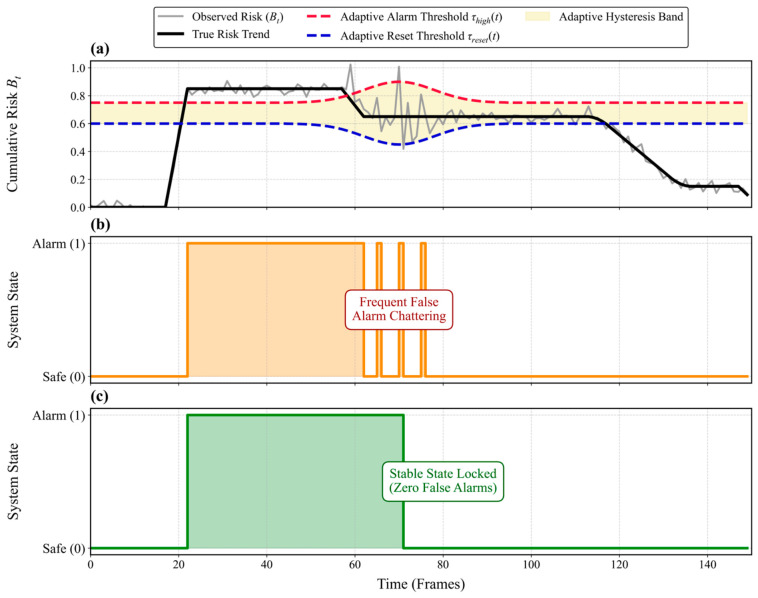
Hysteresis filtering and anti-noise analysis of ST-FSM. (**a**) Risk signal evolution with uncertainty-aware adaptive ST-FSM thresholds; (**b**) output system state without hysteresis, demonstrating severe chattering under high noise; (**c**) output of the uncertainty-aware adaptive ST-FSM, showing a robust and stable state locked with zero false alarms.

To verify the contributions of each core module, this study designed stepwise superimposition ablation experiments, with the results shown in Table 3. Group 1 acts as the pure YOLO detection baseline, relying solely on bounding box overlap to perform instantaneous decisions. Lacking semantic modeling and spatio-temporal smoothing, its F1-Score is only 80.2%, FAR reaches 18.2 times/hour, and JRS is only 45.2%. It exhibits severe boundary jitter and instantaneous false alarms in dynamic chemical scenarios. Group 2 introduces the heterogeneous scene graph to integrate spatial constraints into the graph topology through virtual environment nodes. The F1-Score improves to 82.1%, FAR decreases to 15.0 times/hour, and JRS rises to 55.0%, indicating that environmental semantic modeling possesses preliminary suppression capabilities against spatial false alarms. Group 3 superimposes pose constraints and utilizes YOLO-Pose hand keypoints to refine the discrimination of “human–object” interactions. The F1-Score improves to 87.5%, FAR decreases to 8.4 times/hour, and JRS rises to 68.1%. This proves that pose information can effectively exclude false-positive samples representing “passing by without operation” and significantly enhance interaction discrimination accuracy. Group 4 introduces the anisotropic dynamic field to transform the “hard threshold” of spatial risk into a “soft gradient”, effectively resolving geometric ambiguities under mobile viewpoints. The F1-Score improves to 90.3%, FAR decreases to 3.5 times/hour, and JRS rises to 84.0%, verifying the suppression effect of soft constraint modeling on boundary jitter and spatial false alarms. Group 5 serves as the complete model, superimposing temporal inference by introducing temporal confidence integration and state machines. The F1-Score reaches 93.1%, FAR decreases to 1.3 times/hour, and JRS reaches up to 92.3%. This demonstrates that temporal smoothing can effectively filter out noise brought by instantaneous occlusion and detection jumps to achieve dual spatio-temporal robustness and accurately identify long-term violation behaviors. The effect is shown in Figure 5. The ablation experiments fully verify the effectiveness and necessity of each core module.

### 4.5. Sensitivity Analysis and Selection Criteria for Key Parameters

For the key hyperparameters in the model, this study discarded the empirical trial-and-error method of conventional deep learning. This research strictly followed the chemical process safety standards (such as HAZOP), ergonomic statistical characteristics, and control system filtering theories to provide an analytical basis for parameter selection:

1. Potential Field Geometric Parameters ($\gamma_{hold}$ and σ): This study set the scale tolerance coefficient $\gamma_{hold}$ to 0.15. This value was derived from the statistical surveying of industrial valves and operation panels in the CIR dataset. Under two-dimensional image projection, 0.15 times the maximum edge length of the operation area precisely encompasses 95% of the legitimate manual operation human tolerance range to effectively filter out false-positive events of personnel “passing by but not contacting”. This work dynamically set the potential decay coefficient σ to the square of the equivalent pixel value corresponding to the physical safety radius of the area (e.g., a 1.5 m isolation zone). This ensures that the risk response of the model decays exponentially to a negligible level (<5%) after exceeding the physical safety distance.
2. Inherent Regional Hazard Coefficient ($\alpha_{z}$): According to the HAZOP (Hazard and Operability Analysis) matrix of process industry standards, this study set $\alpha_{z}=0.8$ for high-risk toxic areas and high-pressure areas (directly associated with the upper limit of the alarm state interval) and set $\alpha_{z}=0.5$ for general operation areas. This ensures that the baseline value of the field strength response strictly matches the authentic physical risk level.
3. Temporal Low-Pass Filtering Factor (λ): This research set λ=0.9. Under the 30 FPS sampling rate of the system, this value corresponds to a discrete time constant of approximately 10 frames (about 0.33 s). This setting mathematically perfectly smooths out the common high-frequency visual target losses of three to five frames brought by the chassis movement of the inspection trolley (due to short-term occlusion or severe jitter), while preventing the system response from exhibiting unacceptable excessive hysteresis when authentic hazards occur.
4. Hysteresis State Machine Threshold Design ($\tau_{high},\tau_{reset}$): This study set the baseline alarm trigger threshold $\tau_{high}=0.75$; the baseline recovery threshold $\tau_{reset}=0.60$. In the background working condition evaluation of the validation set, the risk feature background observation noise variance $\sigma_{v}$, caused by front-end positioning algorithms and lighting variations, was approximately 0.04. The hysteresis margin Δτ=0.75−0.60=0.15 designed in this study satisfies $\Delta \tau >3\sigma_{v}$ (corresponding to the 99.7% confidence interval of a Gaussian distribution). This analytical design effectively reduces the sensitivity of the state transition function to unmeasurable noise perturbations to significantly mitigate the “flickering” dilemma near the critical alarm point.

## 5. Conclusions

To address the difficulties of visual noise and high-frequency false alarms caused by dynamic viewpoints in mobile chemical inspections, this study proposed and verified a dynamic risk inference model integrating spatio-temporal semantic constraints. The research results show that joint spatio-temporal physical constraints possess higher robustness than purely data-driven models under complex industrial non-stationary perturbations. By transforming spatial geometric hard boundaries into continuous risk gradients based on anisotropic dynamic fields and introducing uncertainty-aware adaptive hysteresis filtering temporally to conduct confidence integration, the model theoretically achieves noise-reduction decoupling for spatial blur and temporal jitter. It effectively suppresses the alarm oscillations caused by local high-frequency viewpoint changes and presents a highly viable solution to mitigate the practical engineering problem of unreliable state estimation under mobile viewpoints.

Compared with traditional detectors relying on static overlap degree judgments and implicit end-to-end temporal networks, the model compensates for their lack of physical spatial priors when facing high-risk occlusions and lighting mutations. Multi-condition experimental tests demonstrate that while maintaining a peak F1-Score of 93.1%, the model significantly compresses the system false alarm rate to 1.3 times/h, and the average single-frame processing time is only 24.8 ms. Physical deployment on an industrial edge node (Jetson AGX Orin) demonstrated a real-world processing latency of 42.5 ms/frame, successfully bridging the gap between theoretical spatio-temporal modeling and practical, low-latency mobile robot engineering.

Regarding model generalization, while the current system was validated in a specific coal chemical park, the core mathematical logic—the kinematic anisotropic field and adaptive hysteresis—is agnostic to the specific camera hardware or plant layout. By fine-tuning the YOLOv11 frontend on new proprietary data and updating the global SLAM map, the framework can seamlessly generalize to other industrial setups, such as nuclear power plants or offshore oil rigs, accommodating different hazardous-zone definitions.

However, this study still possesses certain limitations. In the current model, the spatial anchoring $C_{z}$ of virtual environment nodes primarily relies on the static prior input of the global map. The model lacks dynamic adaptive capabilities for sudden hazardous sources without fixed boundaries (such as large-area gas diffusion from unknown pipelines). Future research plans to introduce multimodal sensing signals such as gas concentration and infrared thermal imaging to explore the dynamic scene graph growth mechanism of cross-modal feature fusion to achieve complex process safety early-warning with stronger generalization capabilities.

## Figures and Tables

**Table 1:** Comparison of the proposed framework with existing methodologies.

Method Category	Spatial Topology Constraint	Kinematic Awareness	Temporal Filtering Logic	Dynamic Noise Suppression
Traditional Anomaly Detection (e.g., YOLO + Rules)	None (Hard Bounding Box)	None	Instantaneous (No Memory)	Low (Vulnerable to Occlusion)
Static Scene Graph (e.g., MotifNet)	Explicit (Nodes and Edges)	None	Instantaneous	Medium (Topological Jumps)
Implicit Temporal Models (e.g., CNN + LSTM)	Implicit (Black-Box)	None	End-to-End Smoothing	Medium (Lacks Geometric Prior)
Proposed Framework (Ours)	Explicit (Heterogeneous Graph)	Yes (Anisotropic Field)	Adaptive Hysteresis ST-FSM	High (Decoupled Spatio-temporal)

**Table 2:** Performance comparison of different methods on the CIR test set.

Group	Subset-A (F1 (%))	Subset-B (F1 (%))	Subset-C (F1 (%))	Overall FAR (Times/Hour)	Inference Time (ms)
Baseline 1	71.5 ± 2.4	68.2 ± 3.1	75.8 ± 2.8	18.2 ± 3.5	12.5
Baseline 2	78.3 ± 1.8	74.5 ± 2.1	81.0 ± 2.0	8.5 ± 1.8	12.7
Baseline 3	82.1 ± 1.2	80.3 ± 1.5	85.1 ± 1.3	2.3 ± 0.4	35.6
Baseline 4	83.6 ± 1.4	81.2 ± 1.7	85.5 ± 1.5	4.1 ± 0.8	48.2
Baseline 5	88.5 ± 0.9	87.2 ± 1.1	89.0 ± 1.0	1.8 ± 0.3	110.5
Ours	90.8 ± 0.6	91.2 ± 0.8	91.3 ± 0.7	1.3 ± 0.2	24.8

**Table 3:** Ablation study on the effectiveness of core modules.

Group	Basic Scene Graph	Pose Constraint	Anisotropic Field	Temporal Inference	F1-Score (%)	FAR (Times/Hour)	JRS (%)
1	—	—	—	—	80.2	18.2	45.2
2	√	—	—	—	82.1	15.0	55.0
3	√	√		—	87.5	8.4	68.1
4	√	√	√	—	90.3	3.5	84.0
5	√	√	√	√	93.1	1.3	92.3

(Note: The symbol “√” indicates that the corresponding component/method is utilized).

## Data Availability

The datasets presented in this article are not readily available due to strict industrial security regulations and corporate confidentiality agreements from the cooperating chemical enterprise. Requests to access the datasets should be directed to the corresponding author for scientific research purposes only, subject to approval.
